# Assessing the reliability of self-reported weight for the management of heart failure: application of fraud detection methods to a randomised trial of telemonitoring

**DOI:** 10.1186/s12911-017-0426-4

**Published:** 2017-04-18

**Authors:** Adam Steventon, Sarwat I. Chaudhry, Zhenqiu Lin, Jennifer A. Mattera, Harlan M. Krumholz

**Affiliations:** 1 Center for Outcomes Research and Evaluation, Yale School of Medicine, New Haven, USA; 2 Yale School of Medicine, New Haven, USA; 3 Data Analytics, The Health Foundation, 90 Long Acre, London, WC2E 9RA UK

**Keywords:** Telemedicine, End-digit preference, Self-report, Alert fatigue

## Abstract

**Background:**

Since clinical management of heart failure relies on weights that are self-reported by the patient, errors in reporting will negatively impact the ability of health care professionals to offer timely and effective preventive care. Errors might often result from rounding, or more generally from individual preferences for numbers ending in certain digits, such as 0 or 5. We apply fraud detection methods to assess preferences for numbers ending in these digits in order to inform medical decision making.

**Methods:**

The Telemonitoring to Improve Heart Failure Outcomes trial tested an approach to telemonitoring that used existing technology; intervention patients (*n =* 826) were asked to measure their weight daily using a digital scale and to relay measurements using their telephone keypads. First, we estimated the number of weights subject to end-digit preference by dividing the weights by five and comparing the resultant distribution with the uniform distribution. Then, we assessed the characteristics of patients reporting an excess number of weights ending in 0 or 5, adjusting for chance reporting of these values.

**Results:**

Of the 114,867 weight readings reported during the trial, 18.6% were affected by end-digit preference, and the likelihood of these errors occurring increased with the number of days that had elapsed since trial enrolment (odds ratio per day: 1.002, *p <* 0.001). At least 105 patients demonstrated end-digit preference (14.9% of those who submitted data); although statistical significance was limited, a pattern emerged that, compared with other patients, they tended to be younger, male, high school graduates and on more medications. Patients with end-digit preference reported greater variability in weight, and they generated an average 2.9 alerts to the telemonitoring system over the six-month trial period (95% CI, 2.3 to 3.5), compared with 2.3 for other patients (95% CI, 2.2 to 2.5).

**Conclusions:**

As well as overshadowing clinically meaningful changes in weight, end-digit preference can lead to false alerts to telemonitoring systems, which may be associated with unnecessary treatment and alert fatigue. In this trial, end-digit preference was common and became increasingly so over time. By applying fraud detection methods to electronic medical data, it is possible to produce clinically significant information that can inform the design of initiatives to improve the accuracy of reporting.

**Trial registration:**

ClinicalTrials.gov registration number NCT00303212 March 2006.

## Background

Heart failure affects five million people in the United States, [1] and is marked by episodes of acute decompensation with worsening signs and symptoms such as difficulty breathing, leg or feet swelling, and fatigue. Averting these episodes may have a stabilising effect on the progression of the disease, improving quality of life as well as averting costly hospital admissions. Many strategies rely on telemonitoring, whereby patients measure items such as body weight on a regular basis at home and relay that information to physicians working at a distance. Provided the information collected via telemonitoring is accurate, it may have utility to physicians when assessing the need for preventive measures to reduce the risk of acute decompensation.

Telemonitoring services for people with heart failure often emphasise weight monitoring, since a sudden change in weight can indicate fluid build-up, [2] and changes in body weight have been shown to be predictive of hospital admissions for heart failure [3, 4]. Weighing scales have been developed that relay readings automatically to clinical teams (for example, over Bluetooth and home internet connections), without the need for transcription by the patient, but those devices can be costly [5] and are not always preferred by patients [6]. Therefore, simpler approaches have been advocated that allow patients to use existing technology such as standalone weighing scales and telephone keypads [7]. But since those approaches require patients to enter data manually, reporting errors can occur, for example due to problems with memory or low engagement with the monitoring process. These inaccuracies can lead to false alerts as well as missed opportunities to deliver preventive care, adding to the complexity of managing the disease. It is important that clinical teams know how frequently patient-reported data are subject to common forms of error, and the characteristics of patients associated with worse reporting. However, despite the ubiquity of weight monitoring, [2] to our knowledge no studies have examined the accuracy of the patient-reported weights that emerge from telemonitoring systems.

One of the methodological problems in this area is that, in everyday practice, it is not possible to compare weights that are measured by patients at home with a ‘gold standard’, for example weights that are measured contemporaneously by health care practitioners. In this paper, we adapt methods from the fraud detection literature, [8, 9] which are based on theoretical models regarding the distribution of numbers that arise as part of natural processes. Fraud detection methods are appealing because they can be applied to existing data, without additional data collection. Since the accuracy of telemonitoring data will vary between health care systems depending on the details of implementation and local context, our method has been designed to enable local teams to monitor the accuracy of their telemonitoring data. As we describe below, similar methods have been applied in a diverse range of fields, such as forensic accounting and to uncover scientific fraud.

Our case study is a large randomised controlled trial of telemonitoring (the Telemonitoring to Improve Heart Failure Outcomes trial, or Tele-HF), [10] during which 826 patients with a recent hospital admission for heart failure were asked to submit daily weight measurements using their telephone keypads. We focus in particular on numbers that end with certain digits (specifically, 0 or 5), since a tendency towards these digits has been reported across a wide range of areas [11–13]. End-digit preference is common and can reduce the predictive signal from patient-reported weight data – especially since, for patients with heart failure, clinically-significant changes in weight can be as small as two pounds (0.9 Kg) [14–18]. For example, if a patient regularly rounds their weight to the nearest five pounds, then a small change from 142 to 143 pounds from one day to the next would result in a false positive, since in that instance weight would be reported as jumping from 140 to 145 pounds. False negatives can also occur: for example, a large increase in weight from 143 to 147 pounds would register as no change under end-digit preference, even though it is an important factor to consider when prescribing diuretics or other preventive measures. Thus, there are several ways in which end-digit preference can limit the ability of health care professionals to offer timely and effective preventive care.

## Methods

### The Tele-HF trial

The Tele-HF trial recruited 1,653 patients with a recent hospital admission for heart failure from 33 cardiology practices across the United States. Patients were randomised to receive telemonitoring (*n =* 826) or usual care (*n =* 827) for six months [10].

The trial used technology that was familiar to patients. Thus, telemonitored patients were asked to measure their weight daily using a digital scale that was supplied as part of the trial, and to make daily, toll-free calls to the telemonitoring system. During these calls, patients used their telephone keypads to enter their weight as whole number. They also answered questions about signs and symptoms of heart failure, general health and, every thirty days, symptoms of depression. Patients did not have the option to skip a question but could hang up the telephone at any time. Weight was the usually the last information requested within the session. Clinicians reviewed the information as if it was obtained during routine clinical care and might use it, for example, to adjust the dose of diuretic drugs.

### Extent of end-digit preference

Many fraud detection methods have been developed to investigate the plausibility of a data set, within varied fields such as forensic accounting, [8] election fraud, [19] and scientific fraud [9]. These methods are often applications of Benford’s law, which states that, for certain types of data, the leading digits follow a particular distribution, with more numbers beginning with 1 than any other digit [20]. Since weight readings do not follow this pattern, [21] we developed an approach based on the distribution of the final digits, [8, 19] rather than the leading digits. Specifically, we divided the reported weights by five and calculated the remainders – these could be equal to 0, 1, 2, 3 or 4. We reasoned that, if all patients reported their weight accurately, without end-digit preference, then all five remainders are equally likely to occur [22].

We began with a simple analysis, which assessed the number of weight readings affected by end-digit preference, rather than the number of patients affected. We conducted a chi-squared test to assess whether the distribution of the remainders was statistically different from a uniform distribution. We estimated the number of weight readings affected by end-digit preference as the difference between: i) the number of weight readings that ended in 0 or a 5; and ii) the number of such readings that would be expected to arise by chance (*i.e.*, 20% of the total number of weight readings).

We conducted logistic regression to test whether the probability of reporting weight as a multiple of five changed over time during the trial period – this might happen, for example, if patients adhered increasingly poorly to the telemonitoring schedule as time passed. In this regression, we included one observation for each of the weight readings that were reported during the trial period, with our dependent variable being a binary variable relating to whether or not the observed weight was a multiple of 5. The explanatory variable was the number of days since study enrolment.

We checked the specificity of our results by repeating these procedures when dividing by prime numbers other than five. Then, having implemented these relatively straightforward methods, we further developed our approach to estimate the number of patients who were affected by end-digit preference (as opposed to the number of readings), and the characteristics of those patients.

### Estimating the number of patients with end digit preference and their characteristics

We hypothesised that there were two groups of patients: those with a preference for numbers ending in 0 or 5 and those without such a tendency. These groups were referred to as the End-Digit Preference (EDP) and No End-Digit Preference (NEDP) groups, respectively. In the absence of a gold standard, EDP patients could not be identified directly from the data set, so we developed a two-stage method to estimating their number and characteristics. In the first stage, we identified a set of patients that was likely to be a superset of the EDP group: in other words, it contained the vast majority of EDP patients but also some NEDP patients. We refer to this superset as ‘A0’, for the reasons described below. Then, in the second stage of the analysis, we estimated the number of EDP patients by subtracting from the number of patients in A0 an estimate of the number of NEDP patients contained within A0. A similar approach was applied to estimate the characteristics of EDP patients. Below, we describe the method in more detail.

We began by identifying telemonitoring patients who reported weights ending in 0 or 5 more frequently than would be expected by chance (hence the name ‘A0’: these patients tended to report weights that had a remainder of 0 after being divided by five). Not all of these patients had end-digit preference, since sometimes the true weight is a multiple of five, and where a patient’s weight is also relatively stable, a large proportion of their measurements could be multiples of five even without end-digit preference. Thus, A0 will contain some NEDP patients. Nevertheless, we show that our approach to defining A0 identifies the vast majority of EDP patients. To determine whether or not a given patient should be assigned to A0, we restricted our attention to the weight readings submitted by that patient, and calculated the percentage of the remainders that were equal to 0. That percentage was compared to 20% using the binomial proportion test. Any patient whose (one-sided) *p-*value was below 0.05 was assigned to A0. This decision rule is arbitrary – we could have used a different statistical test or a higher or lower *p-*value threshold, but we chose the binomial proportion test and a threshold of 0.05 since they demonstrated some desirable qualities in initial simulations (see Appendix A and B).

In the second stage of the analysis, we repeated the procedure by selecting patients who disproportionately reported a weight that had a remainder of 1, 2, 3, or 4, again by applying binomial proportion tests separately to the weights submitted by each patient – we referred to these subgroups as A1-A4, respectively. We used A1-A4 to estimate the number and characteristics of NEDP patients in subgroup A0. The intuition is that NEDP patients are equally likely to be assigned to any of the five subgroups A0-A4, and therefore, subject to statistical variation, we would expect similar numbers of NEDP patients in each. Therefore, we estimated the number of NEDP patients in A0 as the average of the number of patients in A1-A4; we then subtracted that number from the total number of patients in A0 to estimate the number of EDP patients. By a similar argument, we estimated the characteristics of NEDP patients in A0 by taking the average across all patients in subgroups A1-A4. Then, the characteristics of EDP patients were estimated using a method based on weighted averages (see Appendix A and B for more information). Confidence intervals were obtained for the characteristics of both groups using bootstrapping, which is a nonparametric statistical approach that relies on forming a large number of replica data sets. The replica data sets contained the same number of observations as the original data set, and were constructed by selecting observations with replacement from the original data. The quantity of interest was re-estimated on each replica data set using the methods described above, and then a confidence interval constructed based on the degree of variation observed across the replica data sets [23].

Our approach should ideally demonstrate two properties. First, EDP patients should have a high probability of assignment to subgroup A0. Second, subgroups A1-A4 should consist exclusively of NEDP patients. A simulation study (described in Appendix A and B) confirmed the plausibility of both assumptions; however, we note here that our assumptions are both conservative. If some EDP patients were not assigned to subgroup A0, then we would have underestimated (rather than overestimated) the number of patients with EDP. Likewise, if subgroups A1-A4 contained some patients with EDP, then we would have underestimated the magnitude of the differences in baseline characteristics between the EDP and NEDP patients.

### Analysis of telemonitoring alerts

In addition to comparing the baseline characteristics of EDP and NEDP patients, we compared the number of alerts to telemonitoring system for these two groups (these alerts were referred to as ‘variances’ in the original trial). We anticipated that EDP patients would trigger more alerts than NEDP patients, due to the extra volatility in measurement induced by inaccurate reporting. One limitation of our method is that the analysis of alerts cannot be adjusted for differences in the baseline characteristics of the two groups, but we present our results to raise hypotheses for future studies and to illustrate the potential implications of EDP.

Alerts were identified retrospectively by applying the algorithm adopted for the trial, [10] which required a change of at least three pounds in either direction from the first recorded weight. We assumed that alerts would not be generated for consecutive days, unless the patient’s weight changed and was still at least three pounds from baseline. We also retrospectively applied another algorithm, under which alerts were raised if weight changed by more than two pounds in either direction from one day to the next.

## Results

Out of the 826 patients who were assigned to telemonitoring, 119 patients either did not activate the telemonitoring equipment or could not be linked to the telemonitoring data for this study. Thus, we studied 707 patients, who submitted a total of 114,867 weight readings across the 180-day trial period (corresponding to 162 readings per patient on average).

**Fig. 1:**
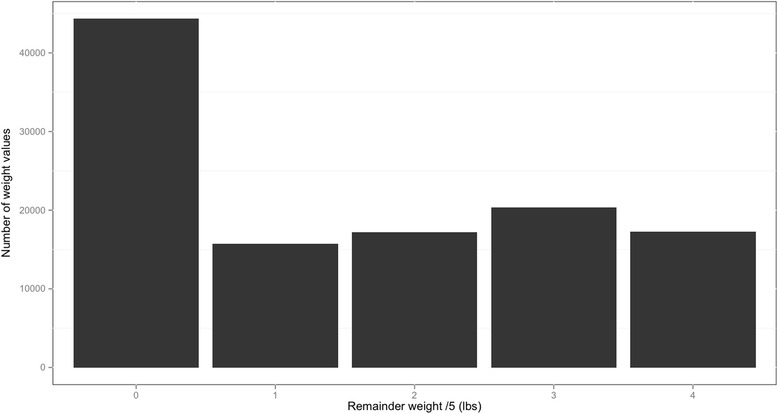
Remainders of weight values reported to the telemonitoring system (*n =* 114,867)

Although we would expect around 22,973 of weight values to be a multiple of five (one-fifth of the total), there were in fact 44,346 such values (38.6%; see Fig. 1). Thus, there were 21,373 more multiples of five than would be expected – implying that 18.6% of all readings were affected by end-digit preference. The chi-squared test confirmed the pattern of remainders was unlikely to be the result of chance (test statistic = 25,350, *p <* 0.001). Logistic regression found that patients were more likely to report their weight as a multiple of five as more time elapsed in the study (odds ratio per additional day since trial enrolment: 1.002, 95% confidence interval 1.001 to 1.002, *p <* 0.001). As anticipated, our results were specific to dividing by five rather than other prime numbers (Appendix A and B).

We identified 212 patients in subgroup A0, compared with 96, 107, 130 and 95 patients in A1, A2, A3 and A4, respectively. Since the mean number of patients across subgroups A1-A4 was 107, we estimated that there were at least 105 EDP patients (*i.e.*, at least 14.9% of patients were subject to EDP). Systematic differences were found in the characteristics of patients in A0 versus the other subgroups (see Appendix A and B). By applying weighted averages, we estimated that, compared with NEDP patients, EDP patients were younger (mean age 60.1 *vs.* 64.0), more likely to be male (68.6% *vs.* 55.2% of patients), less likely to have hypertension (63.4% *vs.* 77.0%), more likely to have graduated high school (82.0% *vs.* 75.6%), and more likely to be in the ‘other’ racial category (*i.e.*, non-white and non-black, 27.3% *vs.* 4.9%) - see Table 1. EDP patients also had lower baseline weight (165.6 *vs.* 172.4 pounds) than NEDP patients and received more of each of the five categories of medication. However, the 95% confidence intervals for EDP patients overlapped with those for NEDP patients for every variable apart from race.

**Table 1:** Baseline characteristics of patients with and without end-digit preference. Data show percentages or mean values (95% confidence intervals)

	End-digit preference (*n =* 105)	No end-digit preference (*n =* 535)
Mean age in years	60.1 (56.3 to 64.0)	64.0 (62.6 to 65.3)
Male	68.6 (55.1 to 83.0)	55.2 (50.7 to 59.6)
New York Heart Association class		
I	2.6 (0 to 8.3)	4.0 (2.3 to 5.8)
II	43.6 (30.1 to 56.9)	35.5 (31.0 to 40.2)
III	45.2 (30.8 to 59.8)	52.2 (47.4 to 56.5)
IV	8.6 (0.6 to 17.6)	8.4 (5.8 to 11.2)
Race		
White	54.2 (39.4 to 67.6)	58.4 (54.2 to 62.4)
Black	18.5 (6.0 to 32.1)	36.7 (32.5 to 41.1)
Other	27.3 (18.1 to 37.6)	4.9 (3.0 to 7.2)
Hispanic or Latino ethnic group	0 (0 to 3.6)	3.0 (1.6 to 4.7)
LVEF < 40%	75.7 (62.4 to 88.5)	65.8 (61.2 to 70.3)
Chronic kidney disease	59.9 (44.4 to 74.2)	56.8 (52.3 to 61.5)
COPD	19.6 (8.6 to 31.3)	20.9 (17.3 to 24.8)
Diabetes mellitus	52.8 (38.9 to 67.9)	52.2 (47.4 to 56.5)
Hypertension	63.4 (50.6 to 76.8)	77.0 (73.4 to 81.1)
Coronary artery disease	55.1 (41.4 to 69.3)	60.1 (55.6 to 64.5)
Mean blood pressure		
Systolic	122.2 (115.3 to 129.0)	121.4 (119.1 to 123.8)
Diastolic	69.7 (65.8 to 73.7)	70.2 (69.1 to 71.5)
Mean serum potassium	4.0 (3.9 to 4.2)	4.1 (4.1 to 4.2)
Mean blood urea nitrogen	31.9 (26.4 to 38.2)	28.1 (26.4 to 29.9)
Mean serum creatinine	1.6 (1.4 to 1.9)	1.5 (1.4 to 1.6)
Mean weight in lbs.	165.6 (142.9 to 187.1)	172.4 (165.8 to 179.4)
Medications		
ACE inhibitor or ARB	73.5 (59.3 to 87.5)	66.1 (61.7 to 70.6)
Aldosterone-receptor antagonist	41.6 (29.6 to 55.2)	31.2 (27.1 to 35.5)
Beta blocker	87.0 (76.2 to 96.7)	82.3 (78.5 to 85.7)
Digoxin	30.2 (17.1 to 42.3)	24.5 (20.3 to 29.0)
Loop diuretic	82.5 (71.4 to 93.9)	80.1 (76.2 to 83.6)
Graduated high school	82.0 (70.0 to 94.4)	75.6 (71.5 to 79.6)
Household income < $10,000 pa	23.6 (9.9 to 37.8)	22.4 (18.1 to 26.8)

As anticipated, EDP patients were more likely to trigger alerts than NEDPs, regardless of alerting algorithm (Table 2). For example, using the definition of alerts from the trial, EDP patients had 2.9 alerts on average during the six months of the trial, compared with 2.3 for NEDPs (Table 2). The weight values reported by subgroup A0 showed greater variability than those reported by the four other subgroups, with a mean within-person variance of 4.73 (versus 3.57, 4.44, 4.20, and 4.42).

**Table 2:** Alerts triggered by patients with and without end-digit preference during the six-month trial period (95% confidence intervals)

	End-digit preference (*n =* 105)	No end-digit preference (*n =* 535)
Mean number of alerts (Tele-HF)^a^	2.9 (2.3 to 3.5)	2.3 (2.2 to 2.5)
Mean number of alerts (2 pound)^b^	3.2 (2.6 to 3.7)	2.6 (2.4 to 2.7)

^a^A difference of more than three pounds in either direction from the first-recorded weight

^b^A difference of more than two pounds in either direction from one day to the next

## Discussion

Health care practitioners rely on self-reported weights to make treatment decisions for patients with heart failure, and inaccurate data will add to the complexity of managing the disease. However, within the Telemonitoring to Improve Heart Failure Outcomes trial, almost one-fifth (18.6%) of weight values were inaccurate due to end-digit preference, and these inaccuracies became more frequent as the trial progressed (*p <* 0.001). A pattern emerged that patients with end-digit preference were younger, more likely to be male, more likely to have graduated high school, and less likely to be in white or black racial groups than other patients. A larger study is needed to confirm these differences between the characteristics of EDP and NEDP patients, since in this study the 95% confidence intervals overlapped for every variable apart from race.

These findings are concerning because clinically-significant changes in weight can be as small as two pounds for patients with heart failure, meaning that rounding can result in both false positives and false negatives. Indeed, we observed that patients with end-digit preference generated an average of 2.9 alerts to the telemonitoring system over the six-month trial period (95% CI, 2.3 to 3.5), compared with 2.3 for other patients (95% CI, 2.2 to 2.5), even though the baseline characteristics of the end-digit preference group did not indicate higher clinical risk. It is possible that the extra volatility induced by measurement error led to more false positives amongst the group with end-digit preference, in turn potentially leading to unnecessary treatment and ‘alert fatigue’ on the part of health care practitioners [24]. We also observed that  end-digit preference became more common over time, which might indicate that patients engaged less with the monitoring process as time wore on. To improve the situation, telemonitoring services that use self-reported data will need to be complemented with efforts to improve the accuracy of reporting, and the methods presented in this paper could be applied to local data sets to determine how to target these interventions on certain population groups.

Further research is necessary to establish the mechanism by which self report errors occur and to test hypotheses regarding which groups of patients it is more likely to affect. Although we used fraud detection techniques, it is important to recognise that there are many possible explanations for our findings, and not just deliberate rounding by patients. For example, during the Tele-HF trial, anecdotal reports emerged that some patients with visual impairments found the digital weighing scales difficult to use. Other explanations to consider are that patients were not able to recall weight measurements accurately, or that patients were reluctant to weigh themselves frequently (perhaps because they perceived that the process unnecessarily reminded them of their health conditions or because they did not believe the intervention to be effective) [25]. Such subjects may have approximated their weight to satisfy the input method for the study, particularly as there was not an option to ‘skip’ a question during the telephone calls.

There is little prior information from the telemonitoring literature regarding which patient characteristics are associated with accurate reporting, though one previous study from a non-telemonitoring setting reported gender differences in whether weight readings were rounded up or down [22]. There may be clues from other areas: for example, studies examining the relationship between patient demographics and the accuracy of self-reported data on service utilisation have produced mixed results, but older age typically emerges as a factor associated with inaccurate reporting [26, 27]. It is possible that hypertensive patients were more familiar with the need to record health status accurately because they already monitored their blood pressure, explaining their lower levels of end-digit preference in this study. Differences by racial group might in part reflect differences in end-digit preference by language [11].

### Strengths and limitations

Our method did not rely on comparisons between patient-reported weight and readings obtained by health care teams, since those comparisons are not usually possible within routine clinical practice. Instead, we identified patients submitting an unusually high number of round numbers, and corrected for chance occurrences to estimate the characteristics of the group of individuals with end-digit preference. However, while our study reflected the usual situation within routine practice, we could not assess the accuracy of individual weight readings, whether patients weighed themselves as instructed, whether weight readings were rounded accurately, or whether there was a systematic tendency to over or under report weights.

Since our sample was recruited from 33 cardiology practices across the United States, we expect it to be broadly representative of the population with heart failure receiving telemonitoring in routine settings. However, the patients examined were receiving an active intervention, which may have influenced the reported values, [28] and perhaps improved reporting relative to routine practice.

This study used a balance of conventional and more innovative techniques. We adopted a relatively straightforward method to estimate the total number of weight readings that were subject to end-digit preference, which was based on dividing the reported weights by five and comparing the resultant distribution with the uniform distribution. Our method for determining the number of patients with end-digit preference and their characteristics was necessarily more sophisticated, and it relied on two assumptions: first, that all patients with end-digit preference were contained within a specific subgroup of patients (‘A0’); and second, that none of those patients were present in four other subgroups (‘A1-A4’). Our simulations confirmed that these assumptions were plausible, but in any case our method is conservative. Thus, we have produced a lower bound for the number of patients with end-digit preference, which might have exceeded 105 (or 14.9% of the total). In particular, we may have missed ‘occasional rounders’ and people who had strong end-digit preference but submitted relatively few readings. These considerations mean that the differences in patient characteristics between EDP and NEDP groups might differ than those reported in Table 1, and likewise for the alerting rates in Table 2. As we note above, the confidence intervals reported for EDP patients in Tables 1 and 2 overlapped with those for NEDP patients, so a larger study is needed to produce more definitive information about which patient characteristics are associated with end-digit preference. Other methods could also be pursued, such as those that model end-digit preference as a latent variable. Finally, we note that this study examined end-digit preference, but other forms of self-report error may exist.

Problems with self-report error are well recognised across a range of other areas, [29] although previous studies have not examined data from telemonitoring systems, or used fraud-detection techniques [11–13]. Our method can be applied to quantify end-digit preference in telemonitoring services for other conditions, such as blood pressure monitoring for hypertension [7]. The method might also be used to test the accuracy of information recorded in the electronic medical record, such as blood pressure and heart rate.

## Conclusion

Our study can be considered an example of ‘failure analysis’, [30] since it retrospectively examines data collected for an intervention that did not produce its intended outcomes, and considers an element that was presumed to be precondition for its success (namely, the ability to collect reliable data from patients on their body weight using existing technology). We found that end-digit preference was prevalent in the Tele-HF trial, affecting almost one-fifth of readings. However, it is unclear to what extent these inaccuracies accounted for the failure of the telemonitoring intervention tested within the Tele-HF trial to reduce hospital readmissions and mortality [16]. Health care practitioners may have been able to compensate for errors in the data when making decisions. Additionally, there can be multiple points of failure for complex interventions. Telemonitoring requires several other elements, including approaches to patient selection, predictive algorithms, and preventive care. Another randomised controlled trial reported no association between telemonitoring and hospital admissions, even though weights were transferred automatically rather than using self-reports [31].

Since end-digit preference can lead to both false positives and false negatives and potentially contribute towards alert fatigue, our results have implications for the design of telemonitoring services. The problem may be concentrated among patients who are in certain groups and thus one response is to develop targeted interventions to educate those patients about the need for accurate transcription of data (for example, through reminder messages within the telephone calls). These initiatives could be targeted using analyses similar to those presented here and, indeed, one of the advantages of our method is that could be applied to existing data sets held by individual clinics. Additional encouragement may be required as time wears on. Another approach to addressing reporting error is to use specialised telemonitoring peripherals, but the implications for patient engagement and cost would need to be assessed. Regardless of the approach, our findings lead us to emphasise the need to be mindful of bias when using self-reported data within clinical practice.

## Appendix A: Simulation analysis

### Introduction

In paper, we describe our approach to estimating the number of EDP and NEDP patients and the differences in their baseline characteristics.

To estimate the number of EDP patients, we subtracted the average number of patients in subgroups A1-A4 from the number of patients in subgroup A0. In other words, if subgroup A0 contained *n*_*o*_ people, and likewise for subgroups A1-A4, then we calculated the number of EDP patients as:

$$ \raisebox{1ex}{${n}_0-{\displaystyle {\sum}_{i=1}^4}{n}_i$}\!\left/ \!\raisebox{-1ex}{$4$}\right. $$

The number of NEDP patients was estimated as the number of patients remaining in A0, once the EDPs had been removed, plus the number in subgroups A1-A4.

We estimated the baseline characteristics of NEDP patients as the average across patients in subgroups A1-A4. Then, we estimated the baseline characteristics of EDP patients by taking weighted averages. For example, if the average age of subgroup A0 was *x*_*o*_ years, and likewise for subgroups A1-A4, then we calculated the average age of EDP patients according to the following formula:

$$ \frac{\ {n}_o{x}_o-\raisebox{1ex}{${\displaystyle {\sum}_{i=1}^4}{n}_i{x}_i$}\!\left/ \!\raisebox{-1ex}{$4$}\right.}{\raisebox{1ex}{${n}_0-{\displaystyle {\sum}_{i=1}^4}{n}_i$}\!\left/ \!\raisebox{-1ex}{$4$}\right.} $$

This method relies on the two assumptions:

1. EDP patients have a high probability of being assigned to subgroup A0.
2. Subgroups A1-A4 consisted exclusively of NEDP patients.

Assumption (1) is important to avoid underestimating the number of patients with EDP, since otherwise some EDP patients might be lost to the analysis. Assumption (2) ensures that subgroups A1-A4 provide an estimate of the number and characteristics of NEDP patients in subgroup A0. Although we could not directly verify either of these assumptions from the data, we conducted preliminary simulations to investigate their plausibility, which are described below.

#### Summary of the findings from the simulations

The simulations described below show that assumption 1 is likely to hold provided patients submitted weight readings on a minimum number of days (broadly, 20 days out of the possible 180 days in the trial period). This was indeed the case, but patients with both EDP and very poor levels of compliance to telemonitoring will be underrepresented in our empirical findings. The simulations indicated that assumption 2 is likely to hold provided that there is a minimum level of variability in the underlying weights. The required level of variability was relatively low, broadly requiring that a patient had at least a 5% chance of seeing a change in weight of 1 pound or more on any given day. If, on the other hand, the weight of some patients came from a distribution that is more stable than this, then our findings would be biased towards the null: in other words, the differences between the characteristics of EDP and NEDP patients would be understated.

### Methods for the simulation study

#### Data generating process

The simulations used a random walk process to generate weight data for a large number of hypothetical individuals with heart failure (n = 10,000). The data generating process assumed that the change in a person’s weight from one day to the next was normally distributed. The mean daily weight change was assumed to be 0 (*i.e.*, no systematic growth or reduction in weight over time), while various scenarios were considered for the standard deviation of the daily weight change (0.05, 0.1, 0.5 and 1 pounds). Our main set of simulations assumed that patients submitted weight readings on 162 days during the six-month trial period, the average seen for telemonitoring patients in the trial, but we also conducted other scenarios (10, 20, 40, 60, 80 and 180 days).

Having generated the weight progressions, we randomly assigned a certain proportion of the patients to the end-digit preference group. Various scenarios were considered for the percentage of patients with this preference (15%, 30%, and 60%). Patients with end-digit preference were assumed to apply these preferences on a certain proportion of days, and again various scenarios were considered for how often this occurred (30%, 50% and 70% of days). The probability of an EDP patient expressing their preference to report weight as a multiple of five was assumed to be independent of their weight.

#### Classifying the simulated patients to EDP and NEDP groups

We applied the procedure described in the main paper to the simulated weight progressions. Thus, for each of the scenarios described above, we examined how likely EDP and NEDP patients were to be assigned to the various subgroups. Specifically, we calculated quantities that enabled us to test the plausibility of the two assumptions described above:

- The proportion of EDP patients who were correctly assigned to subgroup A0, and the proportion of EDP patients who were assigned to any of the subgroups A1-A4. As set out in assumption 1, ideally all EDP patients would be assigned to A0, as this would support the view that our empirical findings will reflect the entirety of this group.
- The proportion of people in subgroups A1-A4 who are NEDP patients. This proportion would ideally be 100% because these subgroups are used to estimate the numbers and characteristic of NEDP patients in subgroup A0 (assumption 2). If subgroups A1-A4 contained some EDP patients, this would bias our results towards the null.

We also report the results of a chi-squared test for the null hypothesis that NEDP patients have an equal probability of assignment to any of the subgroups A0-A4. This is the basis for our symmetry argument.

#### Limiting scenario

Ultimately, subgroup assignment was based on taking the integer part of the reported weight and calculating the remainder after dividing this by 5 (*i.e.*, modulo 5). Thus, as the standard deviation for the change in weight increases, the weight progressions modulo 5 become increasingly similar to a random process whereby successive weight values are sampled independently from the uniform distribution on the values {0, 1, 2, 3, and 4}. We also modelled this limiting scenario, as well as the scenario whereby all weights remain constant over time (*i.e.*, the standard deviation of the daily weight change was 0). Starting values for weight were uniformly distributed on {0, 1, 2, 3, and 4} in all scenarios.

### Results of the simulation study

Table 3 in Appendix shows the results of the simulation for the limiting scenario whereby weight values are sampled independently from the uniform distribution on {0, 1, 2, 3, and 4}. In this scenario, EDP patients had a high probability of being assigned to subgroup A0 (virtually 100%) and subgroups A1-A4 consisted virtually exclusively of patients without end-digit preference (again, virtually 100%). Although it has no consequences for the bias of our ultimate estimates, a significant proportion of NEDP patients were not assigned to a subgroup (70%). There was no evidence that NEDP patients had an unequal probability of being assigned to each of the five subgroups (p-value from chi-squared test greater than 0.10 in all cases).

Table 4 in Appendix shows results when there is some autocorrelation between successive weight readings (*i.e.*, weights were taken from a normal distribution). EDP patients still had a very high probability of being assigned to subgroup A0 (virtually 100%). However, in these scenarios, up to 15% of patients in subgroups A1-A4 were EDP patients (since EDP patients could be assigned to multiple subgroups when the standard deviation for the daily weight change was low). The worse case scenario was when weights were constant over time (*i.e.*, the standard deviation was 0) and the picture steadily improved when the weights became more variable.

Table 5 in Appendix reports the impact of varying the assumption about how often telemonitored patients submit weight readings, on the assumption that weight changes show considerable variability over time (*i.e.*, under the limiting scenario shown in Table 3 in Appendix). In these simulations, 42.6% of EDP patients who submitted readings on only 10 days were assigned to subgroup A0, while 1.9% of EDP patients were assigned to one of subgroups A1-A4. Thus, amongst patients with EDP who submitted weight readings on only 10 days, 55.5% were lost to our analysis. This is to be expected since, in those cases, there was insufficient history to establish patterns of reporting that were consistent with end-digit preference. However, amongst EDP patients who submitted weight readings on 20 days, 96.0% were correctly assigned to subgroup A0, and fewer than 4% were lost to the analysis. Amongst those who submitted weight readings on 40 days, 99.8% were correctly assigned to subgroup A0. Moreover, in all of the scenarios described in Table 5 in Appendix, subgroups A1-A4 consisted almost exclusively of NEDP patients, as desired.

### Discussion

The simulations broadly support the two assumptions made in the empirical work.

*Assumption 1: EDP patients have a high probability of being assigned to subgroup A0.* This assumption was found to be plausible in the scenarios set out in Tables 3 and 4 in Appendix, but Table 5 in Appendix indicates that patients with EDP and a low number of weight readings might have been lost to our analysis. However, on average patients in the Tele-HF trial submitted weight readings on 162 days, whereas our simulations indicated that only 20 readings were required to pick up patients’ end-digit preference. Amongst those with very poor levels of compliance to telemonitoring, patients with EDP will be underrepresented in our empirical findings.

*Assumption 2: Subgroups A1-A4 consisted exclusively of NEDP patients.* In the simulations, this assumption was found to be plausible if patients with heart failure show a minimum level of variability in their weight from one day to the next. The required level of variability was relatively low, corresponding to a standard deviation of 0.5 pounds, implying that weight changed by more than 1 pound on fewer than 5% of days. If there was not much variability in weight, then the simulations suggested that subgroups A1-A4 might contain some patients with EDP, which has the potential to bias the results of our empirical analysis towards the null. However, even if weights were completely constant over time, then NEDP patients would still comprise 85% of people in subgroups A1-A4.

Thus, the simulations broadly support the methods presented in the main paper, though as in any simulation study, we addressed a limited number of scenarios.

**Table 3:** Simulation analysis

Proportion of telemonitored patients with EDP (%)	Probability of EDP patients to demonstrate this preference (%)	Assignment of EDP patients (%)		Assignment of NEDP patients (%)		Proportion of people in A1-A4 with NEDP (%)
		A0	A1-A4	A0	A1-A4	
15%	30%	100.0	0.0	5.9	23.5	100.0
15%	50%	100.0	0.0	6.0	23.1	100.0
15%	70%	100.0	0.0	5.6	23.4	100.0
30%	30%	100.0	0.0	5.7	23.0	100.0
30%	50%	100.0	0.0	5.8	23.1	100.0
30%	70%	100.0	0.0	5.8	22.7	100.0
60%	30%	100.0	0.0	5.8	23.2	99.9
60%	50%	100.0	0.0	6.1	23.0	100.0
60%	70%	100.0	0.0	5.5	23.1	100.0

Note: Assumes patients submitted weight readings on 162 days. Based on 10,000 replications of the simulation experiment

**Table 4:** Simulation analysis – effect of alternative assumptions for weight progression

Standard deviation for daily weight change	Assignment of EDP patients (%)		Assignment of NEDP patients (%)		Proportion of people in A1-A4 with NEDP (%)
	A0	A1-A4	A0	A1-A4	
0 (Weights constant over time)	100.0	79.1	20.9	79.1	85.0
0.05	100.0	83.8	27.8	87.2	85.5
0.1	100.0	83.9	33.2	92.6	86.2
0.5	100.0	10.4	24.8	75.4	97.6
1.0	100.0	0.2	11.0	43.0	99.9
Limiting scenario (from Table 1)	100.0	0.0	5.9	23.5	100.0

Note: Assumes 15% of patients have EDP, 30% of the time. Patients submitted weight readings on 162 days. Based on 10,000 replications of the simulation experiment

**Table 5:** Simulation analysis – effect of alternative assumptions for number of weight readings submitted

	Assignment of EDP patients (%)		Assignment of NEDP patients (%)		Proportion of people in A1-A4 with NEDP (%)
	A0	A1-A4	A0	A1-A4	
10	42.6	1.9	3.5	12.9	97.5
20	96.0	4.7	8.4	34.1	97.6
40	99.8	0.6	4.4	17.4	99.4
60	100.0	0.3	4.5	16.9	99.7
80	100.0	0.3	6.7	25.9	99.8
162 (from Table 1)	100.0	0.0	5.9	23.5	100.0
180	100.0	0.0	6.0	22.6	100.0

Note: Assumes 15% of patients have EDP, 30% of the time, with weights that are uniformly distributed on {0, 1, 2, 3, and 4} modulo 5. Based on 10,000 replications of the simulation experiment

## Appendix B: Baseline characteristics for all subgroups, and sensitivity analyses

**Table 6:** Baseline characteristics of patients in subgroups A0-A4. Data show percentages (numbers of patients) unless stated otherwise

	A0 (*n =* 212)	A1 (*n =* 96)	A2 (*n =* 107)	A3 (*n =* 130)	A4 (*n =* 95)
Mean age in years (SD)	62.1 (13.8)	66.1 (14.1)	65.1 (14.2)	63.1 (14.7)	62.1 (15.2)
Male	61.8 (131)	59.4 (57)	58.9 (63)	50.8 (66)	52.6 (50)
New York Heart Association class					
I	3.3 (7)	3.1 (3)	3.7 (4)	5.4 (7)	3.2 (3)
II	39.6 (84)	34.4 (33)	43.9 (47)	30.8 (40)	33.7 (32)
III	48.6 (103)	54.2 (52)	45.8 (49)	52.3 (68)	56.8 (54)
IV	8.5 (18)	8.3 (8)	6.5 (7)	11.5 (15)	6.3 (6)
Race					
White	56.1 (119)	56.3 (54)	63.6 (68)	56.9 (74)	56.8 (54)
Black	27.8 (59)	36.5 (35)	34.6 (37)	36.9 (48)	38.9 (37)
Other	16.0 (34)	7.3 (7)	1.9 (2)	6.2 (8)	4.2 (4)
Hispanic or Latino ethnic group	1.4 (3)	2.1 (2)	1.9 (2)	4.6 (6)	3.2 (3)
LVEF < 40%^a^	70.8 (148)	71.7 (66)	62.1 (64)	66.7 (84)	62.4 (58)
Chronic kidney disease^b^	58.2 (121)	58.3 (56)	62.3 (66)	50.8 (66)	57.4 (54)
COPD	20.3 (43)	28.1 (27)	21.5 (23)	16.2 (21)	18.9 (18)
Diabetes mellitus	52.4 (111)	54.2 (52)	54.2 (58)	53.8 (70)	46.3 (44)
Hypertension	70.3 (149)	78.1 (75)	76.6 (82)	77.7 (101)	75.8 (72)
Coronary artery disease	57.5 (122)	63.5 (61)	56.1 (60)	61.5 (80)	58.9 (56)
Mean blood pressure (SD)					
Systolic	122.1 (23.8)	122.1 (25.4)	120.1 (22.5)	123.1 (24.6)	121.1 (24.5)
Diastolic	70.1 (13.8)	70.1 (14.3)	70.1 (12.2)	70.1 (13.6)	71.1 (12.7)
Mean serum potassium (SD)^c^	4.1 (0.6)	4.1 (0.5)	4.1 (0.6)	4.1 (0.6)	4.1 (0.5)
Mean blood urea nitrogen (SD)^d^	30.1 (20.3)	27.1 (16.3)	30.1 (16.7)	28.1 (18.8)	27.1 (15.4)
Mean serum creatinine (SD)^e^	2.1 (0.9)	1.1 (0.8)	2.1 (0.9)	1.1 (0.8)	1.1 (0.7)
Mean weight in lbs. (SD)	169.1 (79.6)	175.1 (72.5)	173.1 (76.1)	170.1 (73.2)	173.1 (67.3)
Medications					
ACE inhibitor or ARB	69.8 (148)	64.6 (62)	66.4 (71)	70.0 (91)	62.1 (59)
Aldosterone-receptor antagonist	36.3 (77)	31.3 (30)	29.0 (31)	33.1 (43)	31.6 (30)
Beta blocker	84.4 (179)	84.4 (81)	80.4 (86)	82.3 (107)	82.1 (78)
Digoxin	27.4 (58)	28.1 (27)	20.6 (22)	22.3 (29)	28.4 (27)
Loop diuretic	81.1 (172)	79.2 (76)	79.4 (85)	83.1 (108)	77.9 (74)
Did not graduate high school^f^	21.3 (43)	23.7 (22)	23.5 (24)	26.1 (31)	24.2 (22)
Household income < $10,000 pa^g^	23.1 (39)	22.4 (17)	17.3 (14)	26.9 (28)	21.3 (16)

^a^ *N =* 209, 92, 103, 126, 93

^b^ *N =* 208, 96, 106, 130, 94

^c^ *N =* 204, 95, 104, 128, 93

^d^ *N =* 206, 94, 102, 126, 92

^e^ *N =* 208, 96, 106, 130, 94

^f^ *N =* 202, 93, 102, 119, 91

^g^ *N =* 169, 76, 81, 104, 75

**Fig. 2:**
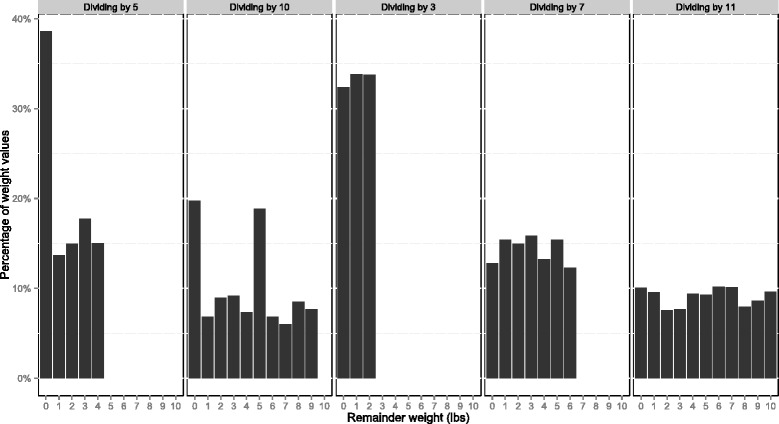
Remainders of weight values reported to the telemonitoring system (n = 114,867)

## Abbreviations

EDP: End digit preference
NEDP: No end digit preference
Tele-HF: Telemonitoring to improve heart failure outcomes
